# Hematocrit and total protein elimination as quality control parameters of cell salvage

**DOI:** 10.1051/ject/2025013

**Published:** 2025-06-16

**Authors:** Debora Dörffel, Edda Klotz, Patrick Wagner, Karsten Ehrig, Karin Pretzel, Falko Hanisch, Axel Pruß

**Affiliations:** 1 Institute of Transfusion Medicine, Charité – Universitätsmedizin Berlin, corporate member of Freie Universität Berlin and Humboldt-Universität zu Berlin Charitéplatz 1 10117 Berlin Germany; 2 Department of Anesthesiology and Intensive Care Medicine, Campus Charité Mitte and Campus Virchow-Klinikum, Charité – Universitätsmedizin Berlin, corporate member of Freie Universität Berlin and Humboldt-Universität zu Berlin Charitéplatz 1 10117 Berlin Germany; 3 Department of Anesthesiology and Intensive Care Medicine, Campus Benjamin Franklin, Charité – Universitätsmedizin Berlin, corporate member of Freie Universität Berlin and Humboldt-Universität zu Berlin Hindenburgdamm 30 12200 Berlin Germany

**Keywords:** Blood transfusion, Autotransfusion, Intraoperative cell salvage, Quality control

## Abstract

*Background*: The use of cell salvage reduces homologous blood transfusions during operations and avoids associated complications. Therefore, autotransfusion is an integral part of Patient Blood Management (PBM). The quality control of cell salvage in Germany is performed by checking the target values of a hematocrit in the autotransfusion blood (target: over 50%) and a total protein elimination (target: over 90%). The aim of this study was to identify intraoperative circumstances leading to deviations from the target values. *Methods*: This retrospective data analysis includes the use of the cell saver at the Charité – Universitätsmedizin Berlin, Campus Charité Mitte and Campus Benjamin Franklin from 01.01.2019 to 29.03.2022, in which autotransfusion occurred. In addition to the hematocrit and total protein elimination, the age and gender of the patients, as well as the surgical diagnosis, were included to investigate possible factors influencing compliance with the target values. The data were statistically analyzed using SPSS. *Results*: A total patient collective of 238 cell salvage applications (154 men, 84 women) was examined. The target values for quality control were achieved on average. The target value for hematocrit was not achieved in about 22% and for total protein elimination in about 8% of cell salvage applications. The age and gender of the patient, as well as the surgical diagnosis, had very little to no influence on compliance with the target values. The target values were not met primarily when the emergency option of the cell saver was used, when the collection volume was too low or when the collection volume was heavily diluted with rinsing fluid. *Conclusion*: The target values for quality control were achieved in most of the cell salvage applications examined and are suitable for ensuring the quality of autotransfusion. Special clinical circumstances may lead to the treating anesthetist having to accept deviating collection volumes or washing processes.

## Introduction

Red blood cell (RBC) concentrates are transfused primarily in cases of acute hemorrhage or chronic anemia to prevent tissue hypoxia [1]. Due to demographic changes with an increase in the proportion of older people with an increasing risk of illness and therefore transfusion, the demand for RBC concentrates will increase in the future [2–4]. In 2010, the WHO recommended the implementation of Patient Blood Management (PBM) [5], which is based on the prevention and early treatment of anemia or coagulopathy, the avoidance of unnecessary blood loss using minimally invasive procedures and the reduction of iatrogenic blood loss, as well as the use of automated autotransfusion and individual decision-making regarding allogeneic blood products, considering patient-specific anemia tolerance [6]. Thus, automated autotransfusion is an integral part of PBM. Allogeneic blood transfusions hold numerous disadvantages compared to autologous blood transfusions: seasonally lower availability, potential transmission of infections, and non-infectious risks. In high-income countries, non-infectious risks predominate [7]. The most serious complications and the majority of deaths are caused by pulmonary complications, such as transfusion-related acute lung injury and transfusion-associated circulatory overload [8]. In addition, allogeneic transfusions can lead to allergic reactions [9]. The rate of adverse reactions to allogeneic transfusions is approximately 1.9 in 1000, of which 43% are allergic transfusion reactions. Although 97% of these allergic transfusion reactions are not severe and are completely resolved by medication, the ongoing transfusion is often canceled and over a third of patients must be transfused again. This not only strains the already scarce supply of blood products but also results in high costs [10]. At the SHOT (Serious Hazards of Transfusion) Symposium 2018, it emerged that over 85% of complications during transfusions were caused by human error in the process, such as misunderstandings, incorrect assumptions, or inadequate handovers [8]. These errors could be reduced by intraoperative cell savers, as the blood product remains in the operating room and therefore no mix-ups can occur. However, the use of intraoperative cell savers is contraindicated in Germany in cases of suspected bacterial contamination of the operating field or bacteremia, as well as in tumor surgery. In contrast to other countries, the use of leucocyte depletion filters alone is not sufficient in Germany for tumor surgery, as the hemotherapy guideline recommends the irradiation of the processed autologous transfusion blood [11]. In Germany, intraoperatively prepared transfusion blood is subject to quality control, whereby the target hematocrit value must be above 50% and the target elimination rate of total protein or albumin must be above 90%. In previous studies, a hematocrit of over 50% of the processed blood could not always be achieved by intraoperative cell salvage [12–15]. Since the recovery of erythrocytes is the main objective of cell savers, the question arises whether an absolute hematocrit target value of over 50% is necessarily relevant for this objective. The hematocrit in autotransfusion blood was particularly low when a small volume of collected blood was processed [13], as the missing volume in the collection bowl must be filled with physiological saline solution before the washing process [14]. The hematocrit in the processed transfusion blood was also lower when using the emergency program, in which the collected blood is processed more quickly, resulting in a lower RBC yield [13, 15]. Nevertheless, the hemoglobin value of patients who receive an intraoperative autotransfusion increases significantly postoperatively [16] and even more than in patients who receive a homologous blood transfusion [17]. Previous studies have shown an average total protein elimination of over 90% [13, 15].

### Study aim

Previous studies have not yet described the influence of patient-dependent factors such as age, gender, and surgical diagnosis on compliance with the target values for the hematocrit value of the prepared transfusion blood and total protein elimination. Similarly, special clinical-surgical circumstances that result in lower hematocrit values have not yet been evaluated, which is the subject of this article. The age and gender of the patients were only recorded to better describe the patient collective under investigation. We did not expect these parameters to have any influence on quality control. Nevertheless, to complete the study, we aimed to investigate the influence of these parameters on total protein elimination and hematocrit.

## Material and methods

Each time an intraoperative cell salvage is performed at Charité – Universitätsmedizin Berlin, a quality control check is carried out. This study includes all uses of the cell savers since January 2019 at Campus Charité Mitte and Campus Benjamin Franklin in which an autotransfusion of the processed blood took place. At Campus Charité Mitte, 107 cases were included until 1 March 2022 and at Campus Benjamin Franklin, 131 cases were included until 29 March 2022. The different end dates result from the different times of data collection at the cell savers. This results in a total patient collective of 238 cases. During the study period, only the 125 mL bowl size was used. The optimal default program (Popt) was used for each wash set. The emergency option was occasionally selected for larger quantities of collected blood in a short period of time. By activating the emergency option, the standard program (Pstd) was selected, which achieves a minimum hematocrit concentration and a minimum wash quality within the shortest possible processing time. The additional options “No Wash” and “Rapid Transfer” in this mode were not selected. The default factory program settings were not changed during usage. Medical cell salvage users were asked about possible reasons for deviations from the target values and acceptance criteria.

This study protocol was reviewed and approved by the Ethics Committee of the Charité – Universitätsmedizin Berlin (approval reference EA2/096/23). The data for quality control of the use of the cell saver is stored in a standard table in the SAP^®^ hospital information system.

### Outcome

The main aim of the evaluation was to assess whether the target values specified in the German hemotherapy guideline of hematocrit (>50%) and a total protein elimination (>90%) in the transfusion blood were not achieved in certain applications of cell salvage, and to investigate the influence of patient-dependent factors such as age, gender, surgical diagnosis, and special surgical circumstances on compliance with the target values.

### Procedures

The cell saver Sorin LivaNova Xtra^®^ is used for intraoperative autotransfusion at the Charité. The device is set up according to the manufacturer’s instructions. The wound blood was aspirated from the surgical site, mixed with the anticoagulant simultaneously, and then stored in a sterile reservoir. Anticoagulation was carried out with heparin (25,000 IU per 0.5 liter of NaCl 0.9%) or alternatively with argatroban (50 mg/L) according to the anesthesiologist’s instructions. If sufficient blood volume was aspirated, the collected blood was transferred and processed in the Latham bowl. As it rotated, the erythrocytes reached the outer wall of the bowl due to their greater density. Plasma, waste components and other blood components, which had a lower density than erythrocytes, then flowed into a waste bag. This washing process continued until the buffy coat reached a certain level, indicating that there were sufficient red blood cells in the bowl. If a bowl could not be sufficiently filled with the available collected blood, the treating anesthesiologist could choose to either fill the rest of the volume with rinsing fluid and accept a low hematocrit, stop the washing process and pump the contents of the bowl back into the reservoir and wait until more collected blood was available, or fill the missing volume with already collected red blood cell concentrate from the retransfusion bag. After this washing process, the concentrated red blood cells were pumped from the bowl into the retransfusion bag. The hematocrit of the collected blood and the processed autotransfusion blood was directly measured by the cell saver using an optical method. The volume of the collected blood and autotransfusion blood was also indicated by the cell saver for each wash cycle, as well as a total volume in each case. Blood samples before and after washing from a randomly selected bowl were collected and analyzed at the Centre for Transfusion Medicine and Cell Therapy Berlin. A sample was taken from the collected blood during the aspiration process to fill the selected bowl. A set for taking blood samples for quality control measurements (MAT-QM-SET, AB 1522, LivaNova^®^) was sterilely attached between the reservoir and the centrifuge bowl and a 50 mL perfusor syringe was connected to the three-way walve. 50 mL of blood was drawn into the perfusor syringe, and the syringe remained connected. A serum tube was then attached to the three-way valve and filled during the filling process. Afterward, 50 mL of blood from the perfusor syringe was returned to the system. A sample was also required from the autotransfusion blood. This was taken during the emptying process after the bowl had been processed. A three-way valve was fitted to the supply tube between the retransfusion bag and the bowl, and the sample in the serum tube was transferred into the retransfusion bag during the emptying process of the bowl. The hematocrit was determined in the laboratory using a Sysmex XS-800i^®^ hematology device. To determine the elimination rate, the total protein in the samples of collected blood and processed autotransfusion blood was analyzed using the Olympus AU480^®^. The elimination rate of total protein was calculated based on the following equation (PB = processed blood; CB = collected blood):

$$\mathrm{Elimination}\;\mathrm{rate}\;\mathrm{of}\;\mathrm{total}\;\mathrm{protein}\;(\%)\;=\;100-\;\left\{\frac{{\mathrm{Total}\;\mathrm{protein}}_{\mathrm{PB}}\times \left({\mathrm{Volume}}_{\mathrm{PB}}\times \left(1\;-\;\frac{{\mathrm{Hematocrit}}_{\mathrm{PB}}}{100}\right)\right)}{{\mathrm{Total}\;\mathrm{protein}}_{\mathrm{CB}}\times \left({\mathrm{Volume}}_{\mathrm{CB}}\times \left(1\;-\;\frac{{\mathrm{Hematocrit}}_{\mathrm{CB}}}{100}\right)\right)}\right\}\;\times 100.$$

### Statistics

The statistical analysis was carried out using the statistical program SPSS version 29.0.1.1 for macOS (IBM SPSS Statistics^®^). Data on hematocrit values in autotransfusion blood and total protein elimination were analyzed separately, but the same statistical methods were used. The descriptive statistics include the number of cases, the minimum, the maximum, the arithmetic means, and the median. The frequency distribution of the values was visualized using histograms. Further tests were carried out to generate hypotheses in an exploratory setting. Scatterplots and Spearman’s correlation were used to further investigate the correlation between the age of the patients and the hematocrit or total protein elimination. The influence of gender on hematocrit and total protein elimination was examined using the Phi coefficient. The influence of surgical diagnosis on hematocrit and total protein elimination was investigated using Cramer-V and the Kruskal-Wallis tests. *P*-values less than 0.05 were considered statistically significant.

## Results

### Enrolment

In 234 of 238 cases, the hematocrit of the processed blood was reported by the cell saver. Of these 234 cases, a diagnosis of surgery was given in 200 cases. In the remaining cases, no surgical diagnosis was entered in the SAP^®^ hospital information system. A total of over 50 different surgical diagnoses were included in this study. The most common surgical diagnoses for which cell salvage was used were aortic aneurysm (49 cases), scoliosis (16 cases), infection and inflammatory reaction due to joint arthroplasty (12 cases), and atherosclerosis of the limb arteries (8 cases). Total protein elimination was reported in 179 of 238 cases. Of these 179 cases, the volumes of collected and processed blood checked on the cell savers matched the data in the hospital information system in 130 cases. This is relevant because the total protein elimination was determined based on the volumes specified in the hospital information system. If the volumes did not match, it must be assumed that an error had occurred during manual data transfer, which is why these cases cannot be included in the quality control. In 110 of these cases, a surgical diagnosis was given.

### Hematocrit of the processed blood

**Figure 1:**
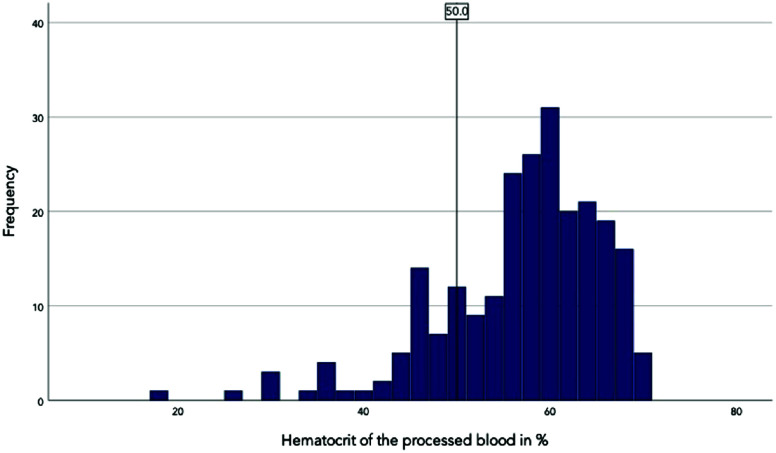
Histogram of the distribution of the hematocrits. The black vertical line in the histogram represents the hematocrit target value of 50%.

**Figure 2:**
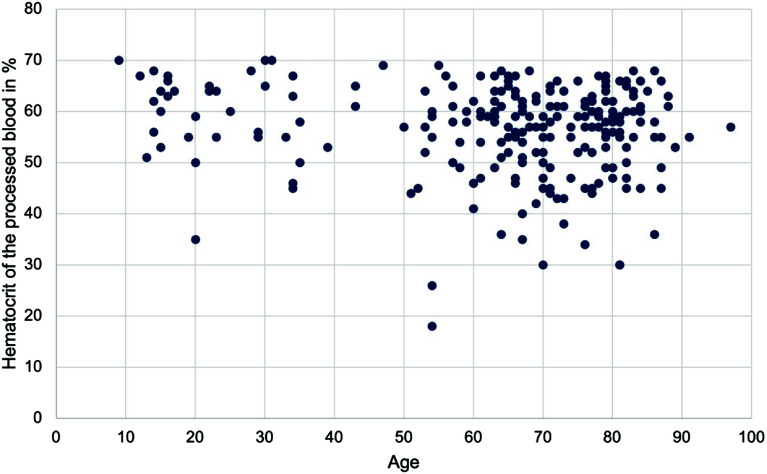
Scatterplot of the age of patients relative to the hematocrit of the processed blood in %.

The distribution of hematocrit values in the processed blood is shown in the histogram (shown in Fig. 1). The hematocrit values were between 18% and 70%. The arithmetic mean was 56.41%, and the median was 58.00%, both of which are within the prescribed target value of 50%. The hematocrit values were not normally distributed. The 25th percentile was at a hematocrit value of 52%, the 50th percentile at 58%, and the 75th percentile at 64%. Of the 234 cases, 52 did not meet the hematocrit target value, corresponding to approximately 22%. In 182 cases, the target value for the hematocrit of the autotransfusion blood was achieved. Of these, 63% were men and 37% were women, which roughly corresponds to the gender distribution of the entire cohort (65% men, 35% women). The mean age of this group was 63 and the median was 69. In 52 cases (22%), the hematocrit of the autotransfusion blood was ≤50%. The proportion of men was 71% and that of women 29%. The mean age was 67, and the median age was 70. No significant correlation was observed between the patient age and the hematocrit value in the processed blood (two-sided *p* = 0.186 by Spearman correlation). The correlation between patient age and the level of hematocrit in the processed blood is shown in a scatterplot, which also indicates no obvious correlation (shown in Fig. 2). Therefore, the patient’s age did not significantly influence the hematocrit. There was also no significant correlation between the patient’s gender and the level of hematocrit in the processed blood (*p* = 0.288 by Phi coefficient). Of the 234 cases in which the hematocrit value was available in the autotransfusion blood, a surgical diagnosis was given in 200 cases. The correlation between the surgical diagnosis and compliance with the hematocrit target was not significant (*p* = 0.147 by Cramer *V*). The distribution of hematocrit in the processed blood did also not differ significantly between the categories of surgical diagnosis (*p* = 0.078 by Kruskal-Wallis-Test). Therefore, there was no significant influence of the surgical diagnosis on the hematocrit value.

### Elimination of total protein

**Figure 3:**
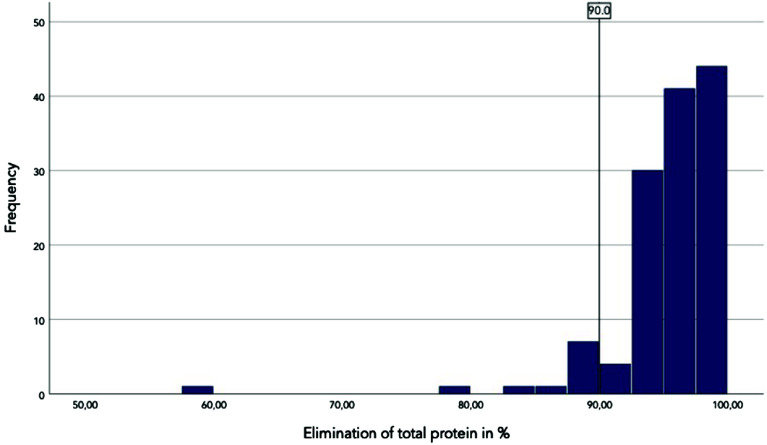
Histogram of the elimination of total protein. The black vertical line in the histogram represents the target value for total protein elimination of 90%.

**Figure 4:**
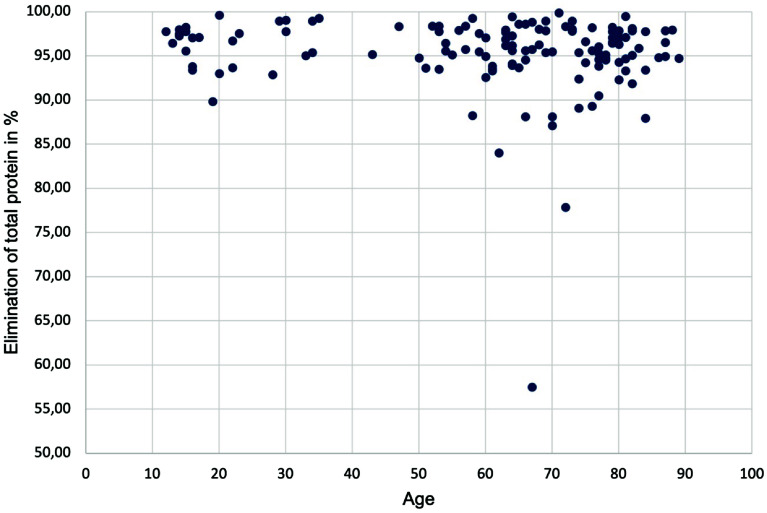
Scatterplot of the age of patients relative to the elimination of total protein in %.

The distribution of total protein elimination is shown in the histogram (shown in Fig. 3). The minimum total protein elimination was 57.5%, and the maximum was 99.9%. The arithmetic mean was 95.24%, and the median was 96.13%, both of which are within the prescribed target value of 90%. The total protein elimination values were not normally distributed. In 11 cases, the target value could not be achieved, corresponding to approximately 8.5%. In the cases where the target value was not reached, the mean age was 65 and the median age was 70, which is slightly higher than in the group that reached the target value (mean age 60, median age 65). No significant correlation was observed between the patient age and the total protein elimination (two-sided *p* = 0.159 by Spearman correlation). The correlation between patient age and total protein elimination is shown in a scatter plot, which also indicates no obvious correlation (shown in Fig. 4). In the group that did not meet the target value, the proportion of male patients was higher, corresponding to 91%. In all cases, regardless of the total protein elimination, the proportion of male patients was around 63%. The Phi coefficient of −0.175 indicates a slight significant correlation between gender and reaching the target value (*p* = 0.046). However, this difference may also be due to the small number of cases in which the target value for total protein elimination was not reached. There was no significant difference in hematocrit distribution of protein elimination between surgical diagnoses (*p* = 0.5 by Kruskal-Wallis-Test). Consequently, we conclude that surgical diagnosis did not have a significant impact on total protein elimination.

## Discussion

Previous clinical studies have not explicitly investigated the influence of patient age, gender, and surgical diagnosis on compliance with the target values for intraoperative cell salvage. This may be because Germany is one of the few countries that have defined target values for intraoperative cell salvage. In this retrospective data analysis conducted at the Charité, it was shown that the target values for hematocrit in autotransfusion blood (>50%) and for total protein elimination (>90%) are not always achieved when intraoperative cell salvage is used. The target hematocrit value was not achieved in around 22% of the cases included. The total protein elimination did not fulfill the target value in 8.5% of cases. The age and gender of the patient and the surgical diagnosis have little to no influence on compliance with the target values.

Identifying the exact factors that contribute to non-compliance with target values for quality control during intraoperative use of cell salvage is not trivial. Various factors influence the course of the operation and, in turn, hematocrit quality and total protein elimination. To retrospectively determine possible causes, the senior physicians responsible for cell salvage at the Department of Anesthesiology and Intensive Care Medicine at the Campus Charité Mitte and the Campus Benjamin Franklin were interviewed.

The main factors that had a negative impact on compliance with both target values were:

1. the use of the cell saver emergency option in the event of massive blood loss and
2. a strong dilution of the collected blood.

The dilution of the collected blood is caused by a high proportion of rinsing fluid relative to a low collection volume, which can be further increased as rinsing fluid is added until the bowl is filled. This often occurs with the last bowl if it is not completely filled. If only a very small volume of collected blood is gathered overall, this also leads to dilution in the first bowl. If the collected blood has fewer red blood cells due to a low collection volume and the remainder of the bowl is filled with rinsing fluid, the processed blood will have a lower hematocrit than undiluted collected blood, despite potentially higher red blood cell yield. Alternatively, processed blood can be taken from the retransfusion bag to fill the remaining bowl and start the centrifugation process. However, the study protocol does not specify which wash cycles this was used for. Therefore, cycles with incompletely filled bowls cannot be excluded from the evaluation. Of the 52 cases in which the hematocrit target value was not reached, the emergency option was used in 6% of cases, whereas a strong dilution effect was identified in 87% (due to one of the reasons already mentioned can be assumed). This means that only in 7% of cases, neither the emergency option nor a dilution effect was likely to be the cause of the deviation from the hematocrit target value. In the remaining cases, the hematocrit in the autotransfusion blood was always ≥45%. Nevertheless, autotransfusion blood with a hematocrit ≤50% was re-transfused, particularly for ethical reasons, if the total red blood cell collection volume was low. Therefore, the hematocrit of the autotransfusion blood can be actively influenced by the anesthesiologist responsible for cell salvage, who may accept deviations at their discretion. Of the 11 cases out of 130 in which the total protein elimination of over 90% was not achieved, 6 cases showed minimal deviations from the target value, with a total protein elimination of over 88%, which could be within the measurement tolerance. In the remaining 5 cases, the emergency option was used in one case, and one had a strong dilution effect. This leaves 3 cases with a total protein elimination below 88% without the reasons already listed. Unlike the hematocrit, the total protein elimination was not determined directly by the cell saver, but only in the laboratory. Therefore, possible sources of error in these cases included sample collection, transport, and laboratory analysis, all of which increased the likelihood of errors in the process of determining laboratory data.

The optimized program (Popt) of the Sorin LivaNova Xtra^®^ cell saver is usually used in our hospital. According to the manufacturer, this achieves a hematocrit in the autotransfusion blood of 60–65% and a total protein elimination of over 95% with a wash cycle duration of 5 min [18]. However, the emergency option can be activated for operations with particularly heavy blood loss. This means that the blood is processed within approximately 3 min, although this results in a lower hematocrit of around 50% in the autotransfusion blood and a total protein elimination of over 92% according to the manufacturer’s specifications [18]. The hematocrit indicated by the sensor of the cell saver Sorin LivaNova Xtra^®^ was compared with the hematocrit values determined by the HemoCue^®^ Whole Blood Haemoglobin System Analyser in a study published by Yarham et al. in 2011. The cell saver Sorin LivaNova Xtra^®^ showed an average hematocrit error of +4% [19]. However, in a further study published the following year, Overdevest et al. [20] concluded that the hematocrit sensor of the Sorin LivaNova Xtra^®^ underestimated the hematocrit in the processed blood by approximately 15% compared to the laboratory device CELL-DYN Sapphire^®^. The hematocrit sensor of the Sorin LivaNova Xtra^®^ was previously used in a study by Melchior et al. [14] for the sole determination of the hematocrit. Several studies have shown that the hemoglobin or hematocrit value of patients increased postoperatively after the intraoperative use of cell salvage, even if the autotransfusion blood itself did not reach the target value of a hematocrit above 50% on average [16, 17, 21]. In addition, the hemoglobin or hematocrit value of the patients who underwent intraoperative cell salvage increased even more postoperatively than in those who received a homologous red blood cell concentrate [17]. The absolute quantity of red blood cells is more important than maintaining the target value hematocrit [22], along with red blood cell yield and, if possible, an increase in the collection volume [21]. Accordingly, it can be concluded that compliance with the hematocrit target value of the autotransfusion blood is not mandatory for the success of the therapy. In addition, deviations in hematocrit can be accepted and actively influenced by the physician in certain clinical situations. Studies addressing the impact of retransfusing cell salvaged blood with less than 90% of total protein elimination are missing.

## Limitation

The present study is a retrospective data analysis. The number of cases and the patient collective were determined by everyday clinical practice. The hematocrit values that were extracted directly from the cell saver are less accurate than the value that were determined by a laboratory device. In addition, the evaluation of total protein elimination was based on a small sample size.

## Conclusion

In the retrospective data analysis presented showed that the target values for total protein elimination and hematocrit in autotransfusion blood were largely achieved during intraoperative use at the Charité Mitte campus and the Benjamin Franklin campus. The determination of total protein elimination with a target value of >90% should remain an integral part of quality control for cell salvage applications, as it is best suited for evaluating the function and suitability of the tested cell saver. However, the quality control requirement for the hematocrit to be above 50% should be reconsidered and could be changed from a mandatory quality assurance value to an optional measurement. The implementation of quality assurance parameters in the intraoperative use of cell salvage varies and only few countries define target values as quality criteria. Therefore, harmonization by international expert committees in anesthesia should be discussed.

## Data Availability

Questions regarding data sharing should be addressed to the corresponding author.
